# Determinants of Early Initiation of Breastfeeding among Mothers of Children Aged Less Than 24 Months in Northwestern Romania

**DOI:** 10.3390/nu11122988

**Published:** 2019-12-06

**Authors:** Anamaria Cozma-Petruţ, Ioana Badiu-Tişa, Oana Stanciu, Lorena Filip, Roxana Banc, Laura Gavrilaş, Daniela Ciobârcă, Simona Codruţa Hegheş, Doina Miere

**Affiliations:** 1 Department of Bromatology, Hygiene, Nutrition, “Iuliu Haţieganu” University of Medicine and Pharmacy, 6 Pasteur Street, 400349 Cluj-Napoca, Romania; anamaria.cozma@umfcluj.ro (A.C.-P.); lfilip@umfcluj.ro (L.F.); roxana.banc@umfcluj.ro (R.B.); laura.biris@umfcluj.ro (L.G.); muresan.daniela@umfcluj.ro (D.C.); dmiere@umfcluj.ro (D.M.); 2 Department of Mother and Child Care, “Iuliu Haţieganu” University of Medicine and Pharmacy, 2-4 Câmpeni Street, 400217 Cluj-Napoca, Romania; badiu.doina@umfcluj.ro; 3 Department of Pharmaceutical Analysis, “Iuliu Haţieganu” University of Medicine and Pharmacy, 6 Pasteur Street, 400349 Cluj-Napoca, Romania; cmaier@umfcluj.ro

**Keywords:** breastfeeding, early initiation, determinants, Romania

## Abstract

Early initiation of breastfeeding (EIBF), defined as putting newborns to the breast within 1 h of birth, may have important benefits for both infant and mother. The aim of this study was to assess EIBF practices and its determinants in northwestern Romania. This cross-sectional study was conducted from March to June 2019, based on a sample of 1399 mothers of children aged less than 24 months. The sample was recruited from the community, from 29 cities and 41 communes distributed across the six counties of the northwestern region of Romania. Mothers responded by face-to-face interviews to a structured questionnaire. Multivariate logistic regression was used to identify factors independently associated with EIBF. Only 24.3% of the mothers initiated breastfeeding within 1 h of birth. Delivering at a private hospital (adjusted odds ratio (AOR): 5.17, 95% confidence interval (CI) 3.87, 6.91), vaginal delivery (AOR: 4.39, 95% CI 3.29, 5.88), mother–newborn skin-to-skin contact for 1 h or more (AOR: 55.6, 95% CI 23.0, 134.2), and breastfeeding counseling during antenatal visits (AOR: 1.48, 95% CI 1.12, 1.97) were factors associated with increased likelihood of EIBF. Overall, the practice of EIBF was poor. Targeting modifiable factors associated with EIBF may be used to improve early initiation practice.

## 1. Introduction

The World Health Organization (WHO) and United Nations International Children’s Emergency Fund (UNICEF) recommend the early initiation of breastfeeding (EIBF), defined as putting newborns to the breast within 1 h of birth [1]. The benefits of EIBF are well-documented. EIBF encourages the attachment between the mother and her child, stimulates milk production, and exhibits positive effects on the establishment and duration of breastfeeding [2]. The first milk after birth is rich in antibodies and other bioactive components that are essential for the immunity, growth, and development of the newborn [3]. There is strong evidence that EIBF is associated with a lower risk of neonatal mortality [4].

Despite the recognized importance of EIBF, only about 50% of the neonates worldwide are breastfed during the first hour of life [5]. As concerns Romania, there are no recent data regarding the practice of EIBF. Previous studies indicated that Romania has one of the lowest rates of EIBF in Europe [6]. In 2002, the Romanian Ministry of Health adopted the WHO/UNICEF-launched Baby-Friendly Hospital (BFH) Initiative. In the same year, the Romanian National Board for Breastfeeding Promotion was established, whose first mission resulted in the elaboration of the Strategy and Plan of Action for Breastfeeding Promotion in Romania for the period 2003–2012 [7]. Nevertheless, in 2004, national statistics showed that only 12% of Romanian mothers started breastfeeding within 1 h of birth [8]. According to a national infant-feeding survey in 2011, Romania had an EIBF rate of 14.8% in the BFH and 5.3% in the other hospitals, respectively [9]. None of these studies assessed the reasons for poor EIBF in Romania. Therefore, there is a need to understand potential barriers towards EIBF in order to help identify and develop appropriate strategies to promote and support timely initiation of breastfeeding in Romania. The aim of this study was to assess EIBF practice and its determinants among mothers of children aged less than 24 months in northwestern Romania.

## 2. Materials and Methods 

### 2.1. Study Design and Setting 

This study was part of a large cross-sectional survey on the nutrition of children aged less than 24 months in northwestern Romania. The survey was conducted between March and June 2019 in the northwestern region of Romania. The region is one of eight development regions in the country and comprises six counties—Cluj, Bihor, Maramureş, Satu Mare, Bistriţa-Năsăud, and Sălaj. The administrative organization of the region consists of 43 cities and 403 communes [10]. As of 1 January 2019 the northwestern region of Romania had an estimated total population of 2,551,234, including 594,391 women in the fertile age range of 15–49 years and 53,756 infants aged 0–23 months [10].

### 2.2. Participants and Recruitment

**Figure 1:**
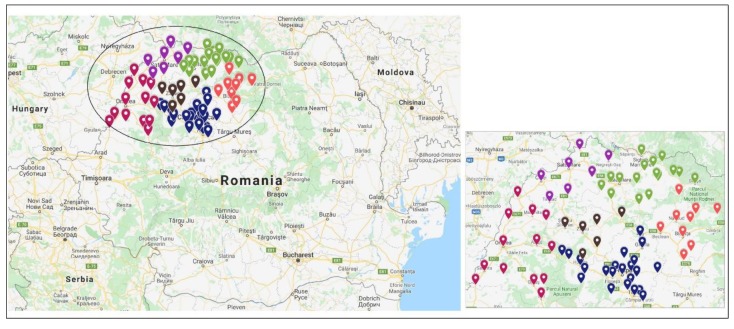
Settlements of sample recruitment, according to county, in northwestern Romania. The map was created with the Google Maps program. Legend: *red dots*—Bihor county; *brown dots*—Sălaj county; *blue dots*—Cluj county; *purple dots*—Satu Mare county; *green dots*—Maramureș county; *orange dots*—Bistrița-Năsăud county.

In the present study, the size of the sample was calculated using Cochran’s formula [11]: $n\;=\;z^{2}pq/m^{2}$ (*n*: sample size; z: standard normal deviate, which is 1.96 at 95% confidence level; p: probability of EIBF (12% according to previous studies [8]); q: 1-p; m: margin of error (5%)). Thus, the minimum sample size required was 162. Data from 1399 mothers were gathered. The sample was recruited from the community, that is, 29 cities and 41 communes across the northwestern region of Romania. Figure 1 displays the geographical range of mother recruitment. Mothers were recruited by advertising the study in nurseries, playgrounds, and child health centers. Mothers were eligible if they were: residents in the northwestern region, if their infants were aged 0 to less than 24 months, were not affected by severe congenital malformations or chronic diseases, and were known to have no dietary restrictions. The mothers who met the inclusion criteria were approached in person and invited to a face-to-face interview after they had received proper oral and written information about the study. Written informed consent was obtained from all mothers who accepted to participate in the study. The study protocol was in compliance with the Declaration of Helsinki, and it was approved by the Research Ethics Committee of the “Iuliu Hatieganu” University of Medicine and Pharmacy Cluj-Napoca, Romania (Approval no. 74/11.03.2019).

### 2.3. Data Collection

Data were collected using a structured questionnaire. Consenting mothers were able to respond to the questionnaire by face-to-face interview, which was conducted by trained data collectors. The questionnaire was developed by reviewing previous research done on a similar topic [12,13,14,15,16,17,18,19] and with guidance from the WHO standardized questionnaire for infant and young child feeding indicators [20].

A pilot survey was conducted in order to test the questionnaire for functionality and clarity prior to distributing it. The pilot survey was carried out among mothers (*n* = 20) in two randomly selected cities in the northwestern region of Romania, and the participants of this survey were excluded from the final study sample. The questionnaire was modified based on the pretesting results. The modifications included rephrasing some questions to improve understandability.

The questionnaire consisted of 65 questions, divided into four main parts. The first part asked general information about the mothers (age, place of residence, marital status, education, occupation, family financial wellbeing) and their infant (gender, age). The second part focused on biomedical data, including parity, gestational age at delivery, place of delivery, and mode of delivery. The third part consisted of questions regarding breastfeeding practice. The fourth part focused on aspects concerning complementary feeding practice. Data provided by questions from the first three parts of the questionnaire were used for the present study.

Therefore, the time of breastfeeding initiation was measured by maternal self-report. The following question was asked to mothers: “How long after birth did you first put your child to the breast?” The responses were as follows: immediately after birth; less than 1 h; between 1 and 24 h; and more than 24 h.

### 2.4. Data Analysis

Data analysis was performed using STATA version 16 (StataCorp. 2019. Stata Statistical Software: Release 16. College Station, TX: StataCorp LLC, USA). Descriptive statistics were performed using frequencies and proportions for independent variables (sociodemographic data, biomedical data). Univariate logistic regression analysis was carried out to determine the strength of association between independent variables and the indicator EIBF. Variables that showed significant association with EIBF at the univariate logistic analysis were included in the multivariate regression model. Adjusted odds ratios (AOR) of significantly associated variables with 95% confidence intervals (CI) have been reported. A resulting *p*-value of less than 0.05 was considered significant for all statistical procedures.

EIBF was assessed based on the WHO indicators for assessing infant and young child feeding practices [20]. EIBF was calculated according to the following Equation:

$$EIBF\;=\;\;\frac{\mathrm{children}\;\mathrm{born}\;\mathrm{in}\;\mathrm{the}\;\mathrm{last}\;24\;\mathrm{months}\;\mathrm{who}\;\mathrm{were}\;\mathrm{put}\;\mathrm{to}\;\mathrm{breast}\;\mathrm{within}\;1\;\mathrm{hour}\;\mathrm{of}\;\mathrm{birth}}{\mathrm{children}\;\mathrm{born}\;\mathrm{in}\;\mathrm{the}\;\mathrm{last}\;24\;\mathrm{months}}\;\times 100$$

## 3. Results

### 3.1. Characteristics of the Respondents

Table 1 presents the socio-demographic and biomedical characteristics of the participants. Out of a total of 1399 mothers, approximately 67% were aged 25 to 34 years, and most (98.8%) were married or living with a partner. The majority of the mothers (69.8%) had a bachelor’s degree or higher, and 88% were employed. Most of the respondents were living in urban areas (73.4%). More than half (59.5%) of the mothers were primiparous, and almost equal proportions had male infants (51.2%) and female infants (48.8%), respectively. Only a low proportion of infants were born pre-term (12.1%). At the time of interviewing, 27% of the infants were below 6 months old, 29% of the infants were 6–11 months old, and 44% were 12–23 months old.

Seventy-three percent of all the mothers delivered at public hospitals, and the proportion of caesarean sections was slightly higher (51.5%) than the proportion of vaginal deliveries (48.5%). While only 28.6% of the mothers attended antenatal birth and baby care classes, half received breastfeeding counseling during antenatal visits. The majority of mothers (75.2%) received postnatal breastfeeding counseling from a healthcare professional, but only a low proportion (21.6%) had skin-to-skin contact with their newborn immediately after delivery.

### 3.2. Time of Initiation and the Early Initiation of Breastfeeding

In the present study, 24.3% of the respondents (*n* = 340/1399) had early initiation of breastfeeding. Table 2 shows that 11.6% of mothers put the child to the breast immediately after birth and 12.6% less than 1 h after birth, respectively. Nevertheless, 47.3% of mothers put the child to the breast between 1 and 24 h, and 28.4% of them put the child to the breast more than 24 h after birth.

### 3.3. Reasons for Delaying the Early Initiation of Breastfeeding and Prelacteal Feeding

The most frequently cited reasons for delaying the EIBF were that “the newborn was taken over by the neonatology service for routine care and evaluation” (583/1059, 55.0%) and “I was under the effect of anesthesia” (419/1059, 39.6%). Other reasons for not breastfeeding within the first hour after birth included that “I had no energy to hold the baby in my arms” (57/1059, 5.3%). Furthermore, as reported in Table 3, 60.2% of the newborns had been fed infant formula as the first feed. More importantly, only 38.7% of the newborns have been fed colostrum, whereas 1.1% received breast milk from another mother.

### 3.4. Determinants of the Early Initiation of Breastfeeding

Table 4 presents the univariate and multivariate logistic regressions examining the factors associated with EIBF. In the multivariate logistic regression analysis using variables that were significant at *p* < 0.05 at the univariate analysis, the place of delivery, mode of delivery, skin-to-skin contact after delivery, and antenatal counseling on breastfeeding were independently associated with EIBF.

Mothers who gave birth at a private hospital were more likely to initiate early breastfeeding compared to mothers who gave birth at a public hospital (AOR: 5.17, 95% CI 3.87, 6.91; *p* < 0.001). There was an increase in the odds of EIBF among the mothers who had a vaginal delivery (AOR: 4.39, 95% CI 3.29, 5.88; *p* < 0.001) than those who had a caesarean section. The odds of EIBF was also higher among mothers who received breastfeeding counseling during antenatal visits (AOR: 1.48, 95% CI 1.12, 1.97; *p* < 0.001) than those who did not receive the counseling during antenatal visits.

Mothers who had direct contact with their newborn after birth, either in the form of skin-to-skin contact for 1 h or more (AOR: 55.6, 95% CI 23.0, 134.2; *p* < 0.001), skin-to-skin contact for less than 1 h (AOR: 4.96, 95% CI 3.52, 6.99; *p* < 0.001), or having the swaddled newborn placed in their arms (AOR: 2.27, 95% CI 1.58, 3.24; *p* < 0.001) were more likely to practice EIBF compared to those to whom the newborn was brought and were only allowed to kiss.

## 4. Discussion

In the present study, only about a quarter of the mothers (24.3%) initiated breastfeeding within 1 h of birth, which is comparable with studies done in other European countries, such as the Republic of North Macedonia (21%) and Montenegro (25.2%) [6]. In contrast, this finding is higher than studies done in Bulgaria (4.6%) and Serbia (7.6%), but lower than studies done in Bosnia and Herzegovina (42.3%), Albania (42.9%), Republic of Moldova (61%), Ukraine (65.7%), Luxembourg (66.5%), Austria (78.1%), and the UK (81%) [6,21,22,23,24]. Nevertheless, the EIBF rate of 24.3% in our study is higher than the 12% reported by the Romanian Ministry of Health in 2004 [8]. Likewise, the result is higher than the average of 8.3% reported by a national infant feeding survey conducted in Romania in 2011, which calculated EIBF as the proportion of children born in the last 12 months who were put to the breast within 30 min of birth [9]. The increase in EIBF practice in Romania could be the result of a series of governmental and community-based interventions to promote EIBF and breastfeeding in general, including many activities in the 2007 World Breastfeeding Week which promoted the importance of EIBF [7,25]. However, according to WHO criteria, the rate of EIBF reported by this study is considered “poor” (range 0–29%) [26]. Therefore, more resources from both national and international organizations are required to promote good breastfeeding practices in Romania.

The main reason reported by the mothers for delaying EIBF was the delay due to the separation of mother and child immediately after birth (55%). Similar findings have been reported by previous studies [27,28]. This delay suggests inadequate practices at the place of delivery and highlights the importance of implementing adequate hospital policies to support EIBF. Such policies should also include discouraging the use of breast milk substitutes. Unfortunately, in this study, 60.2% of mothers reported infant formula as the first feed of their newborn. Prelacteal feeding may weaken the infant suckling stimulus and affect not only the initiation, but also the duration of breastfeeding [29,30,31].

The results of the multivariant analysis indicated that factors significantly associated with EIBF practice were the place of delivery, the mode of delivery, the skin-to-skin contact between the mother and her newborn immediately after delivery, and the breastfeeding counseling during antenatal visits. In contrast to previous studies, EIBF was not significantly associated with maternal age [32], place of residence [22,33,34], marital status [16], education [13,14,28,32,35], occupation [14,35], financial wellbeing [14,24,32,36], parity [34], gestational age at delivery [12,13,37], or postnatal breastfeeding counseling [38].

In Romania, a country with upper-middle income, maternity care is mainly provided by public hospitals and is cost-free [39,40]. In addition, there is an increasing number of private maternity hospitals, which assist women that pay for service fees directly out of pocket or through private health insurance. In the present study, mothers who gave birth at a private hospital were more likely to initiate early breastfeeding than mothers who delivered at a public hospital. Interestingly, this finding is in contrast with a study conducted in Greece [27], a country in which the health system shares similarities with the Romanian health system, both offering public cost-free medical services and private, paid medical services as an option. However, the finding suggests a trend towards an important differentiation between public and private hospitals in Romania. Currently, private hospitals offer high-quality services and tend to be more careful with the patient’s needs than public hospitals. Therefore, in private maternity hospitals, healthcare professionals are immediately present to counsel and support the mother in the initiation of breastfeeding. This may not always be the case in public hospitals, which can be underfunded, overcrowded, and understaffed [41]. In agreement with previous studies, one of the major barriers towards breastfeeding counseling in public hospitals is associated with the excessive workload of healthcare professionals [42]. Likewise, healthcare professionals may lack education and training on breastfeeding, leading to a lack of knowledge and skills required for efficient breastfeeding promotion and support [41,43,44].

Furthermore, the WHO set a value of 15% as the upper limit for medically necessary caesarean sections [45]. However, worldwide, the rate of caesarean sections has increased from an average of 13% in 2005 to more than 20% in 2017 [1]. Worrying results have also been shown by the present study, which reported the rate of caesarean sections as being 51.5%. The study also indicated that mothers with vaginal delivery were more likely to initiate early breastfeeding as compared to those who underwent a caesarean section. This finding is consistent with previous studies conducted in European countries, as well as other countries worldwide [5,16,18,23,46,47]. The delay in initiation of breastfeeding in the case of caesarean sections may be attributed to the practice of separating the newborn from the mother immediately after the surgical delivery, but also to post-caesarean pain and to the hormonal particularities of the caesarean delivery [48]. Studies suggest that additional support in the immediate post-partum period is required for women who undergo caesarean sections in order to help them start breastfeeding as early as possible [49]. Moreover, when the health status of both mother and newborn allow, encouraging skin-to-skin contact as soon as possible after caesarean section has been reported as an effective approach to help mothers establish successful breastfeeding for the first time [50].

In this study, mothers who engaged in early skin-to-skin contact with their newborn were more likely to breastfeed in the first hour after delivery than their counterparts. This finding is similar to previous studies indicating that skin-to-skin contact is associated with improved EIBF practice [24,34,51]. In addition to benefits in terms of EIBF, skin-to-skin contact also exhibits several other benefits, such as contributing to the thermal regulation of the newborn, reducing infant stress, and improving maternal–newborn bonding [52]. Despite the positive effects of skin-to-skin contact, nowadays it has become a common practice to separate mothers from their infant immediately after delivery to perform routine care [52]. However, according to the American Academy of Pediatrics, many postpartum routine procedures can be done while the newborn is experiencing skin-to-skin contact or can be postponed until after the practice of skin-to-skin contact, as long as the mother and child are in a good state of health [53]. Besides, early skin-to-skin contact and initiation of breastfeeding represent Step 4 of the “Ten Steps to Successful Breastfeeding” promoted by the WHO/UNICEF Baby-Friendly Hospital Initiative [54].

In the present study, mothers who were counseled on breastfeeding during antenatal visits were more likely to initiate early breastfeeding than those who did not receive this counseling. This finding is in agreement with previous studies which showed that breastfeeding counseling during antenatal appointments improves the practice of early initiation [12,13,14,18,28,55]. These results may be due to the fact that antenatal visits during pregnancy provide a good opportunity for mothers to acquire adequate knowledge about breastfeeding and understand the benefits of this practice [56].

The present study may have some limitations. Recall bias is a possible limitation in that some mothers were asked to recall an event that occurred up to two years before the study. However, a two-year recall period is the standard recommended by the WHO [20]. Moreover, previous studies indicated that maternal recall of breastfeeding initiation provides a valid and reliable estimate for recall periods of up to three years [57]. This study may also have the limitation that it only reports on EIBF in northwestern Romania. Therefore, the results may not apply to all of Romania. A larger study including mothers from all regions of the country is required to assess the current practice of EIBF in Romania. Additionally, future quantitative and qualitative studies are needed to analyze in more detail the determinants that can lead to successful early breastfeeding. Despite the limitations, the large size of the sample can be considered a strength of the study. Moreover, to our knowledge, this study is the first that reports on EIBF practice and determinants in northwestern Romania, which helps to complete the scarce data regarding EIBF in Europe [6].

## 5. Conclusions

The key finding of this study was that EIBF practice in northwestern Romania is poor according to the WHO criteria. The study also confirmed factors such as delivering at a private hospital, vaginal delivery, mother–newborn skin-to-skin contact, and breastfeeding counseling during antenatal visits as determinants of EIBF. Improving the practice of breastfeeding initiation within the first hour of life is required. Targeting modifiable factors associated with EIBF will allow for policymakers and stakeholders to develop and conduct tailored education programs and supportive interventions for both mothers and healthcare professionals to improve EIBF practice at a national level. Particular interest should be given to facilitate EIBF among mothers who undergo a caesarean section, as this mode of delivery is rising rapidly in Romania.

## Figures and Tables

**Table 1:** Sociodemographic and biomedical characteristics of the participants (*n* = 1399).

Characteristic	Frequency	Percent
Sociodemographic data		
**Maternal age (years)**		
<18	31	2.2
18–24	184	13.1
25–34	940	67.2
≥35	244	17.4
**Place of residence**		
Urban	1027	73.4
Rural	372	26.6
**Marital status**		
Married/Living with a partner	1382	98.8
Single/Divorced/Widowed	17	1.2
**Education**		
≤Secondary school	118	8.4
Completed high school or equivalent	304	21.7
Bachelor’s degree or higher	977	69.8
**Occupation**		
Employed	1231	88.0
Student	89	6.3
Unemployed	79	5.6
**Family financial wellbeing ^#^**		
Poor	437	31.2
Fair	301	21.5
Good	309	22.1
Very good	172	12.3
Excellent	180	12.8
**Infant gender**		
Female	683	48.8
Male	716	51.2
**Infant age (months)**		
0–5	377	26.9
6–11	405	28.9
12–23	617	44.1
**Biomedical data**		
**Parity**		
Primiparous	833	59.5
Multiparous	566	40.5
**Gestational age at delivery (weeks)**		
<37	170	12.1
≥37	1229	87.9
**Place of delivery**		
Public hospital	1021	73.0
Private hospital	378	27.0
**Mode of delivery**		
Vaginal delivery	678	48.5
Caesarean section	721	51.5
**Contact with the newborn after delivery**		
Newborn shown from a distance	185	13.2
Newborn brought near the mother and she allowed to kiss him	640	45.7
Newborn swaddled and placed in the arms of mother	271	19.4
Skin-to-skin contact, for less than 1 h	245	17.5
Skin-to-skin contact, for 1 h or more	58	4.1
**Breastfeeding counseling during antenatal visits**		
Yes	699	49.9
No	700	50.0
**Antenatal birth and baby care classes**		
Yes	401	28.6
No	998	71.3
**Postnatal breastfeeding counseling**		
Yes	1053	75.2
No	346	24.7

^#^ 202 of mothers refused to answer the question about family financial wellbeing.

**Table 2:** Frequency of the time of breastfeeding initiation (*n* = 1399).

Initiation of Breastfeeding After Birth	Frequency	Percent
Immediately	163	11.6
Less than 1 h	177	12.6
1 ≤ 24 h	662	47.3
>24 h	397	28.4

**Table 3:** Frequency of newborn first feed (*n* = 1399).

Newborn First Feed	Frequency	Percent
Colostrum	542	38.7
Infant formula	842	60.2
Breast milk from another mother	15	1.1

**Table 4:** Factors associated with early initiation of breastfeeding among mothers (*n* = 1399).

Characteristic	Unadjusted OR (95% CI)	Adjusted OR (95% CI)
Sociodemographic data		
**Maternal age (years)**		
<18	0.43 (0.14, 1.29)	-
18–24	1.00	-
25–34	0.92 (0.64, 1.32)	-
≥35	1.01 (0.65, 1.57)	-
**Place of residence**		
Urban	1.07 (0.81, 1.41)	-
Rural	1.00	-
**Marital status**		
Married/Living with a partner	1.00	-
Single/Divorced/Widowed	1.71 (0.62, 4.66)	-
**Education**		
≤Secondary school	1.04 (0.63, 1.69)	-
Completed high school or equivalent	1.00	-
Bachelor’s degree or higher	0.96 (0.71, 1.30)	-
**Occupation**		
Employed	1.00	-
Student	0.82 (0.48, 1.38)	-
Unemployed	0.70 (0.39, 1.26)	-
**Family financial wellbeing**		
Poor	1.00	-
Fair	0.81 (0.55, 1.20)	-
Good	0.72 (0.48, 1.06)	-
Very good	0.80 (0.51, 1.25)	-
Excellent	0.80 (0.51, 1.25)	-
**Infant gender**		
Female	1.00	-
Male	1.08 (0.84, 1.38)	-
**Biomedical data**		
**Parity**		
Primiparous	1.00	-
Multiparous	0.95 (0.74, 1.23)	-
**Gestational age at delivery (weeks)**		
<37	0.50 * (0.32, 0.77)	0.65 (0.40, 1.04)
≥37	1.00	1.00
**Place of delivery**		
Public hospital	1.00	1.00
Private hospital	3.94 ** (3.04, 5.12)	5.17 ** (3.87, 6.91)
**Mode of delivery**		
Vaginal delivery	3.28 ** (2.52, 4.26)	4.39 ** (3.29, 5.88)
Caesarean section	1.00	1.00
**Contact with the newborn after delivery**		
Newborn shown from a distance	0.59 (0.33, 1.03)	0.63 (0.36, 1.12)
Newborn brought near the mother and mother allowed to kiss them	1.00	1.00
Newborn swaddled and placed in the arms of mother	2.22 ** (1.56, 3.16)	2.27 ** (1.58, 3.24)
Skin-to-skin contact, for less than 1 h	5.36 ** (3.83, 7.52)	4.96 ** (3.52, 6.99)
Skin-to-skin contact, for 1 h or more	54.36 ** (22.67, 130.34)	55.62 ** (23.04, 134.23)
**Breastfeeding counseling during antenatal visits**		
Yes	1.75 ** (1.37, 2.25)	1.48 ** (1.12, 1.97)
No	1.00	1.00
**Antenatal birth and baby care classes**		
Yes	1.00	1.00
No	0.71 * (0.55, 0.93)	0.82 (0.61, 1.11)
**Postnatal breastfeeding counseling**		
Yes	1.67 * (1.20, 2.32)	1.36 (0.93, 1.99)
No	1.00	1.00

OR: Odds Ratio, CI: Confidence Interval. * *p* < 0.05, ** *p* < 0.001.

## References

[1] UNICEF, WHO (2018). Capture the Moment-Early Initiation of Breastfeeding: The Best Start for Every Newborn. https://www.unicef.org/publications/files/UNICEF_WHO_Capture_the_moment_EIBF_2018.pdf.

[2] Edmond K.M., Zandoh C., Quigley M.A., Amenga-Etego S., Owusu-Agyei S., Kirkwood B.R. (2006). Delayed breastfeeding initiation increases risk of neonatal mortality. Pediatrics.

[3] Ballard O., Morrow A.L. (2013). Human milk composition: Nutrients and bioactive factors. Pediatr. Clin. N. Am..

[4] Smith E.R., Hurt L., Chowdhury R., Sinha B., Fawzi W., Edmond K.M., Neovita Study Group (2017). Delayed breastfeeding initiation and infant survival: A systematic review and meta-analysis. PLoS ONE.

[5] Takahashi K., Ganchimeg T., Ota E., Vogel J.P., Souza J.P., Laopaiboon M., Castro C.P., Jayaratne K., Ortiz-Panozo E., Lumbiganon P. (2017). Prevalence of early initiation of breastfeeding and determinants of delayed initiation of breastfeeding: Secondary analysis of the WHO Global Survey. Sci. Rep..

[6] Bagci Bosi A.T., Eriksen K.G., Sobko T., Wijnhoven T.M., Breda J. (2016). Breastfeeding practices and policies in WHO European Region Member States. Public Health Nutr..

[7] Wallis A.B., Brînzaniuc A., Oprescu F., Cherecheş R.M., Mureşan M., Dungy C.I. (2011). A structured public health approach to increasing rates and duration of breastfeeding in Romania. Breastfeed. Med..

[8] Romanian Ministry of Health, World Bank, UNFPA, USAID, UNICEF (2004). Reproductive Health Survey: Romania. http://siteresources.worldbank.org/INTROMANIA/Resources/study.pdf.

[9] Nanu M.I., Moldovanu F., Stativă E., Stoicescu S. Evaluating the Effectiveness of Interventions Included in National Nutrition Programs for Children Under 2 Years of Age. https://iomc.ro/uploads/documents/Nutritie-sub-2-ani-raport-final-studiu.pdf.

[10] National Institute for Statistics Romania TEMPO-Online Database. http://statistici.insse.ro:8077/tempo-online/#/pages/tables/insse-table.

[11] Cochran W.G. (1977). Sampling Techniques.

[12] Vieira T.O., Vieira G.O., Giugliani E.R., Mendes C.M., Martins C.C., Silva L.R. (2010). Determinants of breastfeeding initiation within the first hour of life in a Brazilian population: Cross-sectional study. BMC Public Health.

[13] Patel A., Banerjee A., Kaletwad A. (2013). Factors associated with prelacteal feeding and timely initiation of breastfeeding in hospital-delivered infants in India. J. Hum. Lact..

[14] Tang L., Binns C.W., Lee A.H., Pan X., Chen S., Yu C. (2013). Low prevalence of breastfeeding initiation within the first hour of life in a rural area of Sichuan Province, China. Birth.

[15] Duan Y., Yang Z., Lai J., Yu D., Chang S., Pang X., Jiang S., Zhang H., Bi Y., Wang J. (2018). Exclusive Breastfeeding Rate and Complementary Feeding Indicators in China: A National Representative Survey in 2013. Nutrients.

[16] Kambale R.M., Buliga J.B., Isia N.F., Muhimuzi A.N., Battisti O., Mungo B.M. (2018). Delayed initiation of breastfeeding in Bukavu, South Kivu, eastern Democratic Republic of the Congo: A cross-sectional study. Int. Breastfeed. J..

[17] Taha Z., Garemo M., Nanda J. (2018). Patterns of breastfeeding practices among infants and young children in Abu Dhabi, United Arab Emirates. Int. Breastfeed. J..

[18] Belachew A. (2019). Timely initiation of breastfeeding and associated factors among mothers of infants age 0–6 months old in Bahir Dar City, Northwest, Ethiopia, 2017: A community based cross-sectional study. Int. Breastfeed. J..

[19] John J.R., Mistry S.K., Kebede G., Manohar N., Arora A. (2019). Determinants of early initiation of breastfeeding in Ethiopia: A population-based study using the 2016 demographic and health survey data. BMC Pregnancy Childbirth.

[20] WHO, UNICEF, USAID, AED, UCDAVIS, IFPRI (2010). Indicators for Assessing Infant and Young Child Feeding Practices. Part II: Measurement. https://apps.who.int/iris/bitstream/handle/10665/44306/9789241599290_eng.pdf?sequence=1.

[21] Petrova S., Rangelova L., Popivanova A., Ovcharova D., Duleva V. (2010). Current infant breastfeeding practice in Bulgaria and determinants. Bulg. J. Public Health.

[22] Institute of Statistics (2010). Institute of Public Health & ICF Macro. Albania Demographic and Health Survey 2008–2009. http://dhsprogram.com/pubs/pdf/FR230/FR230.pdf.

[23] Government of Luxembourg (2010). Feeding our infants. National Nutrition Survey of Children Aged 4, 6 and 12 Months in the Grand Duchy of Luxembourg. http://www.sante.public.lu/fr/catalogue-publications/rester-bonne-sante/alimentation/etude-alba-2008-alimentation-bebes/index.html.

[24] National Health Service UK (2010). Infant Feeding Survey-UK. https://digital.nhs.uk/data-and-information/publications/statistical/infant-feeding-survey/infant-feeding-survey-uk-2010.

[25] National Institute of Public Health Romania (2019). Analysis of the Situation Generated by the Campaign for Celebration of World Breastfeeding Week. http://insp.gov.ro/sites/cnepss/wp-content/uploads/2019/07/SMAS-Analiza-de-situatie-2019.pdf.

[26] WHO, LINKAGES (2003). Infant and Young Child Feeding a Tool for Assessing National Practices, Policies and Programmes. https://apps.who.int/iris/bitstream/handle/10665/42794/9241562544.pdf?sequence=1&isAllowed=y.

[27] Daglas M., Petoussi V., Dionysiou G., Athanassakis I. (2010). Do maternity hospital practices support Greek mothers’ decision to breastfeed?. Clin. Exp. Obstet. Gynecol..

[28] Ahmed A.E., Salih O.A. (2019). Determinants of the early initiation of breastfeeding in the Kingdom of Saudi Arabia. Int. Breastfeed. J..

[29] Häggkvist A.P., Brantsæter A.L., Grjibovski A.M., Helsing E., Meltzer H.M., Haugen M. (2010). Prevalence of breast-feeding in the Norwegian Mother and Child Cohort Study and health service-related correlates of cessation of full breast-feeding. Public Health Nutr..

[30] Hailemariam T.W., Adeba E., Sufa A. (2015). Predictors of early breastfeeding initiation among mothers of children under 24 months of age in rural part of West Ethiopia. BMC Public Health.

[31] Azzeh F.S., Alazzeh A.Y., Hijazi H.H., Wazzan H.Y., Jawharji M.T., Jazar A.S., Filimban A.M., Alshamrani A.S., Labani M.S., Hasanain T.A. (2018). Factors associated with not breastfeeding and delaying the early initiation of breastfeeding in Mecca region, Saudi Arabia. Children.

[32] Esteves T.M., Daumas R.P., Oliveira M.I., Andrade C., Leite I. (2014). Factors associated to breastfeeding in the first hour of life: Systematic review. Rev. Saude Publica.

[33] National Statistical Service, Ministry of Health & ICF International (2012). Armenia Demographic and Health Survey, 2010. http://dhsprogram.com/pubs/pdf/FR252/FR252.pdf.

[34] Singh K., Khan S.M., Carvajal-Aguirre L., Brodish P., Amouzou A., Moran A. (2017). The importance of skin-to-skin contact for early initiation of breastfeeding in Nigeria and Bangladesh. J. Glob. Health.

[35] Adhikari M., Khanal V., Karkee R., Gavidia T. (2014). Factors associated with early initiation of breastfeeding among Nepalese mothers: Further analysis of Nepal Demographic and Health Survey, 2011. Int. Breastfeed. J..

[36] Setegn T., Gerbaba M., Belachew T. (2011). Determinants of timely initiation of breastfeeding among mothers in Goba Woreda, South East Ethiopia: A cross sectional study. BMC Public Health.

[37] Goyal N.K., Attanasio L.B., Kozhimannil K.B. (2014). Hospital care and early breastfeeding outcomes among late preterm, early-term, and term infants. Birth.

[38] Karim F., Khan A.N.S., Tasnim F., Chowdhury M.A.K., Billah S.M., Karim T., Arifeen S.E., Garnett S.P. (2019). Prevalence and determinants of initiation of breastfeeding within one hour of birth: An analysis of the Bangladesh Demographic and Health Survey, 2014. PLoS ONE.

[39] The Constitution of Romania Article 34-Right to Protection of Health. https://www.presidency.ro/en/the-constitution-of-romania.

[40] World Bank (2019). Romania-Health Program for Results. http://documents.worldbank.org/curated/en/318821560866840902/Romania-Health-Program-for-Results.

[41] Coculescu B.I., Coculescu E.C., Purcărea V.L. (2016). Orientation to the patient as a marketing strategy in the Romanian healthcare system. J. Med. Life.

[42] Majra J.P., Silan V.K. (2016). Barriers to Early Initiation and Continuation of Breastfeeding in a Tertiary care Institute of Haryana: A Qualitative Study in Nursing Care Providers. J. Clin. Diagn. Res..

[43] Radzyminski S., Callister L.C. (2015). Health professionals’ attitudes and beliefs about breastfeeding. J. Perinat. Educ..

[44] Gavine A., MacGillivray S., Renfrew M.J., Siebelt L., Haggi H., McFadden A. (2017). Education and training of healthcare staff in the knowledge, attitudes and skills needed to work effectively with breastfeeding women: A systematic review. Int. Breastfeed. J..

[45] WHO, UNFPA, UNICEF, Mailman School of Public Health (2009). Monitoring Emergency Obstetric Care: A Handbook.

[46] Rowe-Murray H.J., Fisher J.R. (2002). Baby friendly hospital practices: Cesarean section is a persistent barrier to early initiation of breastfeeding. Birth.

[47] Sharma I.K., Byrne A. (2016). Early initiation of breastfeeding: A systematic literature review of factors and barriers in South Asia. Int. Breastfeed. J..

[48] Kuyper E., Vitta B., Dewey K. (2014). Implications of cesarean delivery for breastfeeding outcomes and strategies to support breastfeeding. Alive Thrive Tech. Brief.

[49] Hobbs A.J., Mannion C.A., McDonald S.W., Brockway M., Tough S.C. (2016). The impact of caesarean section on breastfeeding initiation, duration and difficulties in the first four months postpartum. BMC Pregnancy Childbirth.

[50] Stevens J., Schmied V., Burns E., Dahlen H. (2014). Immediate or early skin-to-skin contact after a Caesarean section: A review of the literature. Matern. Child Nutr..

[51] Mullany L.C., Katz J., Li Y.M., Khatry S.K., LeClerq S.C., Darmstadt G.L., Tielsch J.M. (2008). Breast-feeding patterns, time to initiation, and mortality risk among newborns in southern Nepal. J. Nutr..

[52] Moore E.R., Anderson G.C., Bergman N., Dowswell T. (2012). Early skin-to-skin contact for mothers and their healthy newborn infants. Cochrane Database Syst. Rev..

[53] American Academy of Pediatrics (2012). Section on Breastfeeding. Breastfeeding and the use of human milk. Pediatrics.

[54] Pérez-Escamilla R., Martinez J.L., Segura-Pérez S. (2016). Impact of the Baby-friendly Hospital Initiative on breastfeeding and child health outcomes: A systematic review. Matern. Child Nutr..

[55] Baker E.J., Sanei L.C., Franklin N. (2006). Early initiation of and exclusive breastfeeding in large-scale community-based programmes in Bolivia and Madagascar. J. Health Popul. Nutr..

[56] Ghimire U. (2019). The effect of maternal health service utilization in early initiation of breastfeeding among Nepalese mothers. Int. Breastfeed. J..

[57] Li R., Scanlon K.S., Serdula M.K. (2005). The validity and reliability of maternal recall of breastfeeding practice. Nutr. Rev..

